# An Innovation to Address Brain Health and Dementia in Individuals with Intellectual and Developmental Disabilities

**DOI:** 10.1002/alz.095609

**Published:** 2025-01-09

**Authors:** Jennifer Pettis, Karen K Tracy

**Affiliations:** ^1^ Gerontological Society of America, Washington, DC USA

## Abstract

Adults with intellectual and developmental disabilities (I/DD) and their caregivers may face many barriers to obtaining an accurate assessment of cognitive decline. For example, primary care providers may mistake manifestations of new cognitive impairments or dementia for symptoms of the underlying disability. Pre‐existing cognitive impairments, communication challenges, unfavorable reactions by members of the care team to adults with pre‐existing cognitive deficits, and lack of understanding regarding common behaviors and needs of adults with I/DD can further complicate assessments. Furthermore, mainstream cognitive screening and assessment tools are not appropriate for many of the more than 7 million people living with I/DD in the United States. To address this unmet need and better equip primary care teams to appropriately address brain health in adults with I/DD, the Gerontological Society of America (GSA) developed a new companion resource to supplement *The GSA KAER Toolkit for Brain Health*.

The four steps in the KAER framework—Kickstart, Assess, Evaluate, and Refer—are intended to improve health‐related outcomes and well‐being for people living with dementia and their families. The Toolkit includes practical approaches, educational resources, and validated clinical tools that help primary care teams implement initiatives related to brain health and timely detection of cognitive impairment. GSA developed the companion publication addressing the unique needs of adults with I/DD. The publication, *Addressing Brain Health in Adults with Intellectual Disabilities and Developmental Disabilities: A Companion to the KAER Toolkit for Primary Care Providers*, aims to:

• Raise awareness of unique needs of adults living with I/DD.

• Equip and encourage caregivers and health care teams to engage in appropriate brain health conversations with adults with I/DD.

• Promote brain health conversations and early detection of changes in cognitive and adaptive function for adults with I/DD.

• Assist with the identification of community supports and resource networks aimed at enhancing function and quality of life for adults with dementia and I/DD.

This presentation will provide insights into the KAER framework and how, when combined with *The GSA KAER Toolkit for Brain Health*, the new companion can support person‐centered brain health promotion, early detection of dementia, and dementia care planning for individuals with I/DD.

